# Seasonality of endemic COVID-19

**DOI:** 10.1128/mbio.01426-23

**Published:** 2023-11-08

**Authors:** Jeffrey P. Townsend, Hayley B. Hassler, April D. Lamb, Pratha Sah, Aia Alvarez Nishio, Cameron Nguyen, Alexandra D. Tew, Alison P. Galvani, Alex Dornburg

**Affiliations:** 1 Department of Biostatistics, Yale School of Public Health, New Haven, USA; 2 Department of Ecology and Evolutionary Biology, Yale University, New Haven, USA; 3 Program in Computational Biology and Bioinformatics, Yale University, New Haven, USA; 4 Program in Microbiology, Yale University, New Haven, USA; 5 Department of Bioinformatics and Genomics, University of North Carolina at Charlotte, Charlotte, USA; 6 Center for Infectious Disease Modeling and Analysis, Yale University, New Haven, USA; 7 Yale College, New Haven, USA; Griffith University-Gold Coast Campus, Gold Coast, Queensland, Australia

**Keywords:** SARS-CoV-2, COVID-19, seasonality, evolution

## Abstract

Successive waves of infection by SARS-CoV-2 have left little doubt that this virus will transition to an endemic disease. Foreknowledge of when to expect seasonal surges is crucial for healthcare and public health decision-making. However, the future seasonality of COVID-19 remains uncertain. Evaluating its seasonality is complicated due to the limited years of SARS-CoV-2 circulation, pandemic dynamics, and varied interventions. In this study, we project the expected endemic seasonality by employing a phylogenetic ancestral and descendant state approach that leverages long-term data on the incidence of circulating HCoV coronaviruses. Our projections indicate asynchronous surges of SARS-CoV-2 across different locations in the northern hemisphere, occurring between October and January in New York and between January and March in Yamagata, Japan. This knowledge of spatiotemporal surges leads to medical preparedness and enables the implementation of targeted public health interventions to mitigate COVID-19 transmission.

**IMPORTANCE:**

The seasonality of COVID-19 is important for effective healthcare and public health decision-making. Previous waves of SARS-CoV-2 infections have indicated that the virus will likely persist as an endemic pathogen with distinct surges. However, the timing and patterns of potentially seasonal surges remain uncertain, rendering effective public health policies uninformed and in danger of poorly anticipating opportunities for intervention, such as well-timed booster vaccination drives. Applying an evolutionary approach to long-term data on closely related circulating coronaviruses, our research provides projections of seasonal surges that should be expected at major temperate population centers. These projections enable local public health efforts that are tailored to expected surges at specific locales or regions. This knowledge is crucial for enhancing medical preparedness and facilitating the implementation of targeted public health interventions.

## INTRODUCTION

The current COVID-19 pandemic has resulted in over 6.5 million deaths worldwide. Public health interventions—especially the closing of schools and universities and banning of large gatherings—were highly effective at reducing transmission at the advent of the pandemic (1). Widespread vaccination further altered the course of the pandemic, saving tens of millions of lives globally in the first year alone (2). However, governmental interventions have ebbed internationally. Sustained transmission is predicted to continue into the foreseeable future (3, 4), and there is now little doubt that COVID-19 is transitioning into a global endemic disease (5). This impending endemicity entails continued surges of infections causing morbidity and mortality that can be mitigated with advance preparation, especially via anticipation of future seasonal infection patterns.

COVID-19 case numbers have fluctuated in different regions and at different times during the last year. However, three challenges impede attempts to directly estimate future seasonal infection patterns from COVID-19 data: global variability in public health measures, evolving pandemic transmission dynamics, and the short duration since SARS-CoV-2 emergence. These factors present too many confounders to yield highly informative studies correlating infection with environmental parameters across locations (6)—parameters such as ultraviolet light, humidity, precipitation, and temperature (7–13). Without well-estimated correlative associations, parameters for epidemiological modeling studies are lacking. With only a few years of infection data collected in the contexts of highly volatile and heterogeneous interventions, there are qualitative inferences but little year-to-year data that can be appropriately applied to determine the seasonality of the virus (14). The absence of annual SARS-CoV-2 infection data without pandemic transmission dynamics or public health interventions has hampered efforts to determine COVID-19 seasonality and resulted in contradictory estimates of seasonal trends (15–17) and necessitates application of alternate approaches.

One approach to predicting SARS-CoV-2 seasonality relies on comparison with other endemic viruses that follow a similar route of respiratory transmission. These “flu and cold” viruses follow known seasonal patterns of infection that vary across the globe (18) and offer a possible analogy to the annual variation expected for SARS-CoV-2 (17, 19–23). However, diverse respiratory viruses exhibit divergent patterns of seasonality. For example, rhinovirus infections occur relatively frequently in April through November, compared to respiratory syncytial virus infections, which are relatively more frequent in December, January, and February (24). Coronaviruses, in particular, may be informative regarding the seasonality of SARS-CoV-2 (25, 26). Yet even at low evolutionary divergences, there is variance in seasonal incidence patterns: across locales in Sweden, coronavirus infection by HCoV-OC43 occurs at its highest frequency in December and January, while infections by HCoV-NL63 generally peak in February (24, 27). As in the case of influenza (28, 29), this seasonal variation is a consequence of evolutionary processes operating on a complex system of human behavior, epidemiology, immunology, and viral genetics.

Prediction of the impending endemic seasonality of SARS-CoV-2 can be performed by leveraging precisely estimated evolutionary divergences between human-infecting coronaviruses, accumulated knowledge of seasonal HCoV coronavirus incidence, and advances in phylogenetic comparative methods that enable the unknown seasonality of SARS-CoV-2 to be estimated. We apply such an approach to the estimation of the seasonality of SARS-CoV-2 infection based on extensive long-term incidence of other coronaviruses (HCoV-OC43, HCoV-NL63, HCoV-HKU1, and HCoV-229E) across major population centers. This analysis provides a means for estimating the seasonal force of infection that is not dependent on isolation of interventions or identification of underlying mechanisms. Our resulting projections of endemic SARS-CoV-2 seasonality provide insight into the optimal long-term public policies that can be applied to high-risk periods and the preparation of healthcare providers for temporally and spatially localized surges.

## MATERIALS AND METHODS

### Study design

We conducted a literature search to identify data on monthly verified cases of HCoV-NL63, HCoV-229E, HCoV-HKU1, and HCoV-OC43 infections within populations across the globe. To infer the seasonality of SARS-CoV-2, we applied ancestral and descendant state analyses on reconstructions of the evolutionary history of human-infecting coronaviruses to estimate the expected annual changes in cases at different geographic locales. These analyses provide a projection of the endemic seasonality for SARS-CoV-2.

### Data acquisition

*Phylogenetic tree topologies*. Phylogenetic relationships of SARS-CoV-2 and HCoVs were based on data from 58 alphacoronavirus, 105 betacoronavirus, 11 deltacoronavirus, and three gammacoronavirus as analyzed by Townsend et al. (30). These estimates of the phylogenetic topology were consistent with previous hypotheses of evolutionary relationships among coronaviruses (31–35) and were congruent across multiple methods of inference with strong (100% bootstrap) support for all nodes. Tree topologies were inferred by multiple maximum-likelihood (ML) analyses of the concatenated DNA sequence alignment, and the results were robust to alternative phylogenetic likelihood search algorithms—IQ-TREE v2.0.6 (36) and RAxML v7.2.8 (37). Results were robust to a potential history of recombination among or within genes through phylogenetic analyses using an alignment of the putative non-recombining blocks (38).

*Phylogenetic branch lengths*. Timetrees were taken from the study of Townsend et al. (30). Briefly, ML phylogenies were time-calibrated using least-squares dating (LSD2; 39) in IQ-TREE v2.0.6 (36). Divergence times were calibrated to the dates of viral sampling associated with the earliest samples of each virus that had been sequenced and deposited in GenBank. Consistency of the divergence time estimates to alternate approaches of divergence time estimation was assessed through comparisons of Relative Times (RelTime; 40) in MEGA X v10.1.9 (41) and TreeTime v0.7.6. The RAxML-derived ML and IQ-TREE-derived ML phylogenies with estimated branch lengths were used as the input phylogeny in RelTime (42) and TreeTime (43). To assess the impact of outgroup choice, TreeTime analyses were repeated with an unrooted input phylogeny and with the option to estimate a root. The resulting relative timetrees were robust to branch length differences arising from different approaches to relative divergence time estimation—IQ-TREE v2.0.6 (36), RelTime (40) in MEGA X v10.1.9 (41), and TreeTime v0.7.6 (43). All trees from Townsend et al. (30) were pruned of tip branches terminating in SARS-CoV-1 and MERS-CoV because temporal trends of infection by these viruses reflect short-term outbreaks and not seasonal endemic circulation.

*Seasonal infection data*. We conducted a literature search using the PubMed and Google Scholar databases searching for terms related to coronavirus, seasonality, and the known seasonal endemic human-infecting coronaviruses (HCoV-NL63, HCoV-229E, HCoV-HKU1, and HCoV-OC43). Searches were conducted in English between October 2020 and August 2021 using the names of each coronavirus lineage as a key term in addition to all combinations of coronavirus, seasonality, environmental, incidence, infection, prevalence, latitude, temperature, humidity, weather, global, and cases—with no language restrictions imposed. A series of searches for additional data in English, Chinese, Japanese, and Spanish language journals were conducted between 30 August and 20 September 2023. Searches were conducted by language speakers and augmented with follow-up searches utilizing ChatGPT v3.5 (OpenAI, 2023) and Google Translate by non-language speakers. Seasonal infection data were extracted from published, peer-reviewed research papers that reported monthly or finer seasonal case data for three or more coronaviruses, spanning at least 1 year.

### Estimating the seasonality of SARS-CoV-2

To estimate the seasonality of infections by SARS-CoV-2, we first extracted the average number of cases per month testing positive for HCoV-NL63, HCoV-229E, HCoV-HKU1, and HCoV-OC43 for each location. We scaled these case counts by the annual total to yield proportions of the cases sampled in each month. We then used Rphylopars v0.2.12 (44) to perform a phylogenetically informed ancestral and descendant state analysis on the monthly proportions of cases to estimate the proportion of yearly infection by SARS-CoV-2 in each month for each location. This approach takes known trait values (here, monthly proportions of cases for endemic coronaviruses) and applies models of trait evolution and a phylogeny to estimate unobserved trait values for a taxon or taxa. A Brownian motion model is commonly applied to phylogenetic evolution of continuous traits (45–48), but other models could also be applied (49). To assess how the specification of a model of trait evolution impacts the resulting inferences of incidence, we repeated the analyses across the range of trait evolution models available in Rphylopars: Brownian motion, Ornstein–Uhlenbeck (OU), Pagel’s lambda, and white noise. The Brownian motion model specifies that trait values evolve over time in accordance with a Gaussian distribution of change. The OU model builds upon Brownian motion by incorporating a parameter for selection drawing lineages toward a fixed value (50, 51). Pagel’s lambda model spans from the Brownian motion model to the white noise model, transforming the internal branch lengths based on the amount of phylogenetic signal of the trait (52, 53). A white noise model provides predictions equivalent to a star tree. Phylogenetic ancestral and descendant analyses were repeated across all topologies resulting from different inference approaches (molecular trees, relative phylogenetic chronograms, and non-recombinant alignment) to assess the impact of phylogenetic inference methods on our estimation of seasonality.

To quantify the relative degree of seasonality among viruses, we calculated the Shannon diversity of monthly proportions for each virus at each site using the vegan package in R (54). Shannon diversity indices were pooled by virus; differences between group means were assessed using an analysis of variance. Pairwise *t* tests were subsequently performed with a Bonferroni adjustment for multiple testing to assess differences in the Shannon diversity of monthly proportions of infections by each virus. Pairwise testing was repeated using Tukey’s honest significant differences, yielding consistent results.

## RESULTS

Our systematic review regarding seasonal patterns of endemic coronavirus incidence identified 19 studies that met the criteria of providing at least 1 year of data on at least three circulating human-infecting coronaviruses within a locale (Table 1). Of these, 12 met a sufficiency of data criterion of having at least 100 cases distributed across the year. These studies spanned three continents across the northern hemisphere (Table 1). In temperate regions, endemic coronaviruses typically exhibited pronounced seasonality (Fig. S1–S4). The seasonal patterns observed in these larger studies were consistent with the results from incidence reports that contained smaller number of samples or limited months of sampling of coronaviruses in locations that include Ishikawa prefecture in Japan (55), Northern Italy (56), and Spain (57).

**TABLE 1:** Data sets on seasonal coronavirus incidence

Data set	Location	Dates^*e*^	Sample*^a^*	HCoV incidence				Reference
				229E	OC43	NL63	HKU1	
North America								
i	Rochester, MN, USA	4/1/2014– 3/31/2020	326	47	103	81	95	(25)
ii	New York City, NY, USA	10/2016– 12/2018	122	31	48	15	28	(58)
—*^b^*	Denver, CO, USA	12/2004– 11/2005	84	11	34	37	2	(59)
United Kingdom								
iii	Edinburgh, United Kingdom	7/2006– 6/2009	267	NA	NA	NA	NA	(60)
Europe								
iv	Stockholm, Sweden	1/2010– 2/2020	2,093	320	1,266	507	*^c^	(61)
v	Trøndelag, Norway	1/2007– 12/2014	263	16	113	84	50	(62)
vi	Gothenburg, Sweden	11/2006– 10/2009	239	33	124	82	*^c^	(24)
vii	Amsterdam, Netherlands	1985– 2011	101	38	30	25	8	(21)
—*^b^*	Tampere, Finland	9/2009– 8/2011	52	13	13	15	11	(63)
Asia								
viii	South Korea*^d^*	1/2010– 12/2012	1,568	153	871	544	*^c^	(64)
ix	Yamagata, Japan	1/2010– 12/2014	388	40	94	154	100	(65)
x	Guangzhou, China	7/2010– 6/2015	293	49	177	44	23	(66)
xi	Sarlahi, Nepal	6/2011– 5/2014	270	19	103	70	78	(67)
—*^b^*	Beijing, China	5/2005– 4/2009	87	15	50	8	14	(68)
—*^b^*	Hong Kong, China	4/2014– 5/2015	87	4	53	17	13	(69)
—*^b^*	Sa Kaeo Province, Thailand	9/1/2003– 8/31/2005	83	13	37	19	14	(70)
—*^b^*	Hong Kong, China	9/2008– 8/2014	77	12	48	6	11	(71)
—*^b^*	Nakhon Si Thammarat, Thailand	7/2009– 6/2010	32	*^c^	22	9	1	(72)
Middle East								
xii	Beersheba, Israel	7/2015– 6/2016	195	10	96	45	44	(73)

^*a*^ Sample numbers may not agree with the study totals summarized in the Results section because some studies included samples that were not associated with coronavirus infection. Also, tabulated numbers in the table may not agree exactly with numbers in tables from the cited papers because some studies exhibited discrepancies between the raw data and their tabulated summaries. In all cases, we used numbers from the available raw data.

^*b*^ These studies were excluded from our primary analysis because they were composed of low sample sizes (<100) across the calendar year. A secondary analysis of these data sets is presented in the Supplementary Materials.

^*c*^ Samples from this region were not assayed for this virus.

^*d*^ Nationwide.

^*e*^ Month/day/year or month/year.

From our literature review, we obtained two data sets pertaining to North America. Data set i contained results from 8,839 nasopharyngeal swabs, bronchoalveolar fluid, or bronchial washes collected between April 2014 and March 2020 from the Mayo Clinic Laboratories in Rochester, MN. Samples were screened for HCoV-229, HCoV-HKU1, HCoV-NL63, and HCoV-OC43 using multiplex respiratory panels (25). Data set ii was composed of 4,215 samples taken from 196 individuals in New York City from October 2016 through April 2018 including children, teenagers, and adults with and without daily contact with children (58). To be included in the data set, cases must have provided nasopharyngeal samples weekly from both nostrils for a minimum of 6 weeks. We obtained five data sets pertaining to Europe. Data set iii included incidence data on HCoV-229, HCoV-HKU1, HCoV-NL63, and HCoV-OC43 from 11,661 respiratory samples from 7,383 patients collected by the Royal Infirmary of Edinburgh between July 2006 and June 2009. Samples were collected as part of routine incidence monitoring from both male and female patients ranging in age from 0 to 3 months to over 65 years (60). Data set iv was composed of 2,084 cases found to be positive for one of the coronaviruses, collected between 1 January 2010 and 31 December 2019 at the Karolinska University Hospital in Stockholm, Sweden (61). Data set v was composed of samples collected at St Olavs Hospital in Trondheim, Norway, from children under 16 years of age who were exhibiting no symptoms and presenting for elective surgery or who were presenting with symptoms of respiratory tract infection. (62). Data set vi was composed of 7,853 samples (239 HCoV-positive) collected from November 2006 to October 2009 from 7,220 patients ranging from age 0 to 98, with a median age of 22, in Gothenburg, Sweden (24). Data set vii was collected from serum and blood samples of adult males in the HIV-1 uninfected cohort of the Amsterdam Cohort Studies on HIV-1 and AIDS at primarily 3-to 6-month intervals spanning a 35-year period (21).

**Fig 1:**
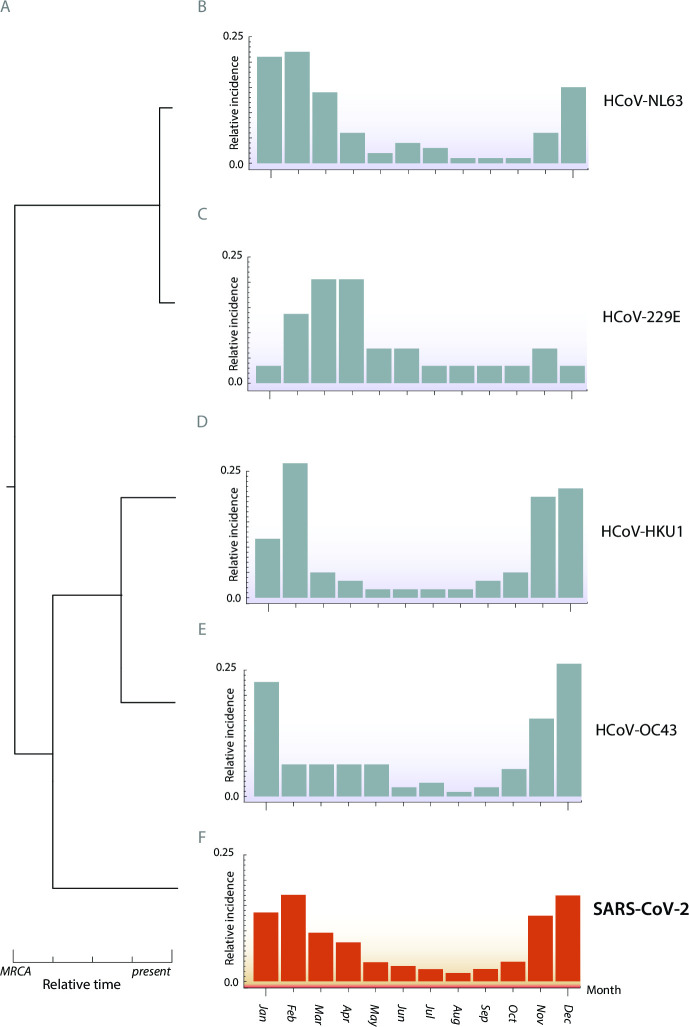
Phylogenetic inference of relative monthly incidence of SARS-CoV-2 under endemic conditions. (**A**) Time tree extending from the most recent common ancestor (MRCA) to current day taxa based on the phylogenetic divergence of HCoV coronaviruses. Empirical relative monthly incidences of (**B**) HCoV-NL63, (**C**) HCoV-229E, (**D**) HCoV-HKU1, and (**E**) HCoV-OC43 and (**F**) ancestral- and descendant-state analytical estimates of relative monthly incidences of SARS-CoV-2 in Trøndelag, Norway.

**Fig 2:**
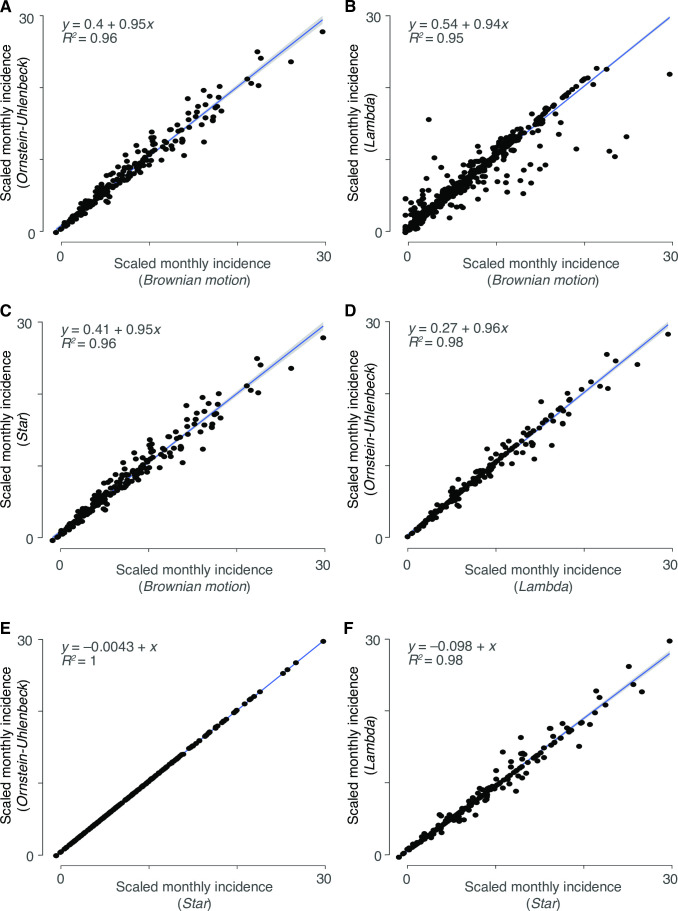
Correlations between models for seasonal projections of all monthly incidences of SARS-CoV-2 infection across all locations. (**A**) OU versus Brownian motion, (**B**) Pagel’s lambda versus Brownian motion, (**C**) white noise versus Brownian motion, (**D**) OU versus Pagel’s lambda, (**E**) OU versus white noise, and (**F**) Pagel’s lambda versus white noise. Monthly incidence estimates were linearly scaled to fit within 0–1 axes and pooled across all location data sets.

We obtained four data sets with sufficient sample sizes pertaining to Asia. Data set viii was collected across 36 facilities in Korea by the Korea Influenza and Respiratory Viruses Surveillance System between 2013 and 2015 via throat swabs of 36,915 patients presenting with symptoms of acute respiratory infections (64). Data set ix was composed of results from throat and nasal swabs of 4,342 patients (3,092 aged ≤5 years, 767 aged 6–10, 326 aged 11–15, and 104 aged >15 years) presenting with symptoms of respiratory infection in pediatric clinics in Yamagata, Japan spanning January 2010 to December 2013 (65). Data set x was sourced from 13,048 throat and nasal swabs of adults and children symptomatic for acute respiratory infection between July 2010 and June 2015 in Guangzhou, China, at an approximately 1.5:1 ratio of males to females (66). Data set xi was composed of results from weekly nasal swabs of 3,693 women enrolled in their second or third trimester of pregnancy, obtained between 2011 and 2014 in the Sarlahi district in Nepal. Participants were enrolled in either their second or third trimester of pregnancy and were monitored until 6 months after giving birth (67). We additionally found a single data set (data set xii) of 195 individuals in Israel who were identified as HCoV-infected in a hospital setting during 2015–2016 (73). Additional data sets with sample sizes less than 100 in each region were retained for additional sensitivity analyses to assess whether results from similar geographic regions were robust to smaller sample sizes. For each location, we pruned the phylogeny of major coronavirus lineages from Townsend et al. (30) to include only the HCoVs with sample data and SARS-CoV-2 (Table 1 and Fig. 1A). To generate ML estimates of the spatiotemporal incidences of SARS-CoV-2, we conducted analyses of ancestral and descendant states on the relative monthly incidences for each coronavirus (Fig. 1B through E). All four endemic coronaviruses contributed to our projection of the relative monthly incidences of SARS-CoV-2 (Fig. 1F). However, the late-diverging HCoV-OC43 and HCoV-HKU1 provide more phylogenetic information than the early-diverging HCoV-NL63 and HCoV-229E. Across cases, estimates of seasonality were strongly correlated between models and nearly interchangeable, indicating the results to be robust to the selected model of trait evolution (Fig. 2).

**Fig 3:**
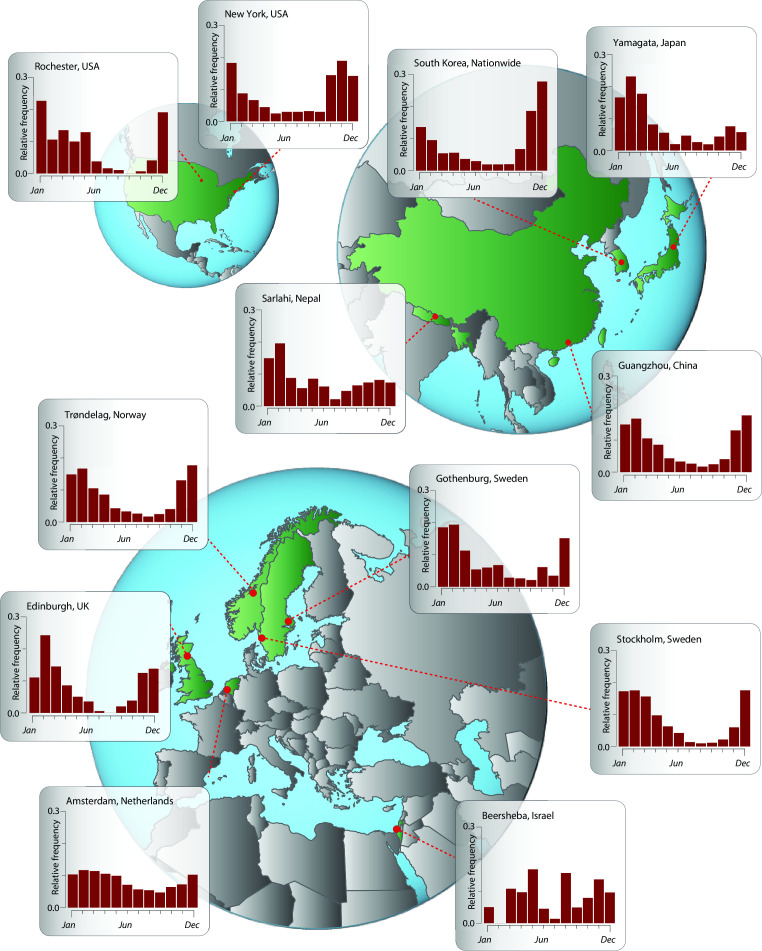
Ancestral- and descendant-state analytical estimates of the relative monthly incidence of SARS-CoV-2 under endemic conditions. New York City and Rochester, USA; Amsterdam, Netherlands; Gothenburg and Stockholm, Sweden; Trøndelag, Norway; Edinburgh, UK; Beersheba, Israel; Sarlahi, Nepal; Guangzhou, China; Yamagata, Japan; and South Korea (nationwide).

Application of this evolutionary analysis to Trøndelag, Norway, provides projections that late fall and winter months will exhibit significantly higher levels of SARS-CoV-2 incidence than summer and early fall months (Fig. 1F). This lower incidence in the summer and surrounding months is largely generalizable to much of the temperate northern hemisphere (Fig. 3). Specifically, significantly higher SARS-CoV-2 incidence is projected in late fall and winter months in New York City . A similar seasonality is projected for multiple locales in Asia, including Yamagata, Japan; Guangzhou, China; and South Korea; as well as Edinburgh, UK; Tampere, Finland; and Gothenburg and Stockholm in Sweden. However, in each northern hemisphere continent, there are regional deviations from this seasonal pattern. In Rochester, incidence is projected not to rise until December, with a prolonged plateau of infection extending through the late spring. Incidence in Amsterdam is similarly projected to decline in late spring, though the overall seasonal trends are more muted than other locations. In Asia, incidence in Sarlahi, Nepal, is projected to surge at the beginning of the new year, while in the Middle East, the seasonality of incidence in Beersheba, Israel, appears atypical with no distinct pattern. In all cases, these results were robust to the phylogenetic inference method, to the underlying molecular data set, as well as to the use of a chronogram or a molecular evolutionary tree (Fig. S5–S8).

Comparison of the Shannon diversities of monthly incidences among coronaviruses at sites revealed that diversity did not vary substantially by coronavirus (Fig. S9). Modest differences in the mean Shannon diversity values among some coronaviruses were weakly supported by an analysis of variance (d.f. = 4, *F* = 2.59, *P* = 0.042). Evaluating pairwise comparisons using a post-hoc Tukey’s Honest significance test did not yield a statistically significant difference in mean diversity across months for SARS-CoV-2 compared to HCoV-NL63 (ratio 1.12:1; *P* = 0.061), HCoV-OC43 (1.12:1; *P* = 0.053), HCoV-229E (0.91:1; *P* = 0.179), and HCoV-HKU1 (1.10:1; *P =* 0.168). Similarly, Tukey’s honest significance test did not yield statistically significant differences in mean diversity across months for any other pairwise comparison of coronavirus at any site (1.00–1.02:1; *P* > 0.98 for all comparisons).

## DISCUSSION

Here we analyzed the monthly incidence data of the long-time circulating coronaviruses HCoV-NL63, HCoV-229E, HCoV-HKU1, and HCoV-OC43 to quantify the seasonality of infection in regions that span a broad range of predominantly temperate localities across North America, Europe, and Asia. We then conducted ancestral- and descendant-state analyses that project the seasonality of SARS-CoV-2 infection as COVID-19 becomes endemic. Across much of the temperate northern hemisphere, SARS-CoV-2 infections can be expected to transition to a seasonal pattern of incidence that is high in late fall and winter months relative to late spring and summer. Our projections also reveal geographic heterogeneity. This heterogeneity often manifested as a syncopation of the general northern hemispheric trend—a delay in rise to peak incidence or a prolonged duration of higher levels of incidence relative to other areas. These temporal transmission patterns of SARS-CoV-2 provide fundamental insights for the determination of local public health policies, enabling preparedness and consequent mitigation of seasonal infection.

Several previous studies have taken on the challenge of predicting seasonality of SARS-CoV-2 based on direct analysis of incidence across seasons during the initial pandemic spread (74–76). During a zoonotic pandemic, immune naïveté, out-of-phase emergence, regional variations in public health intervention, and stochastic pulses of local transmission can obscure the signature of seasonality from surveillance data (14). Such concerns have made these analyses controversial (77, 78). To avoid such concerns, we based our analyses on multi-year long-time circulating coronavirus infection data that were not subject to the biases introduced by pandemic emergence and large-scale public health interventions. Unlike most other studies, our analyses do not force or even suggest any functional form or *a priori* expectation of seasonality. Instead, our results are driven by infection data from other circulating human-infecting coronaviruses and informed by their shared evolutionary history. Results from our analyses are broadly consistent with the seasonal infection trends observed for common respiratory viruses in the northern hemisphere (19).

Our results on the seasonality of SARS-CoV-2 provide expected incidence trends under endemic conditions. Through two alternative mechanisms, seasonality during the pandemic phase of COVID-19 might be either more or less pronounced than our endemic expectations. On the one hand, the absence of previous exposure and the corresponding naïve immune response are associated with higher transmission in a pandemic. This higher transmission would exacerbate the peaks and potentially the troughs of infection. In this context, seasonality can be further amplified by an overwhelmed and lagging public health response. As such, we could observe heightened seasonal differences in incidence relative to those seen during endemic spread, overlaid onto peaks and troughs caused by the out-of-phase emergence of pandemic disease (79). On the other hand, the mechanisms that are driving the seasonality of coronavirus infections might exert slight influences that are magnified by host-pathogen population dynamics year on year (80). This resonation to convergence could underlie the observed seasonality of endemic coronaviruses (Fig. S1–S4). If seasonality in the endemic coronaviruses is a consequence of a small forcing factor that is amplified by host-pathogen population dynamics, then the expectation would be that we would observe less seasonality for SARS-CoV-2 during pandemic spread than would be seen in its eventual endemic incidence. It is likely that not enough time has elapsed for SARS-CoV-2 to completely transition to a stable endemic seasonality. Regardless of how the seasonal dynamics will manifest during this transition from its pandemic phase, our projections provide the expected endemic seasonality.

It is tempting to compare our results to the history of surges throughout the COVID-19 pandemic thus far. For instance, following the initial outbreak, peaks of COVID-19 deaths in Sweden, where interventions were very limited and kept steady, are consistent with our projections of a December–February peak of infection. In much of the rest of the world, however, interventions were more extreme and were unsteadily applied. Relaxation of COVID-19 interventions could explain the “out-of-season” surges of infection in the summer of 2022 in countries such as Japan and irregular patterns of infection in countries that delayed widespread infection such as Australia or New Zealand. Similar irregular patterns can also be found within countries that had variation in interventions such as vaccine uptake or adherence to public health guidelines, including within the United States (81, 82). This range of policies and adherence to guidelines confounds direct comparisons (83).

In addition to heterogeneous health policies, it is also very likely that the urgent rollout of initial vaccination and later waves of booster uptake had substantial effects on the seasonality of COVID-19 during the pandemic phase. Seasonality of circulating coronaviruses HCoV-OC43, HCoV-NL63, HCoV-HKU1, and HCoV-229E—which our predictions are based on, via their evolutionary relatedness to SARS-CoV-2—is not affected by vaccination because there are no currently approved vaccines that target them. Accordingly, substantial uptake of booster vaccinations could alter SARS-CoV-2 seasonality from our endemic predictions. Such an outcome would be expected if boosters are administered to large portions of the global population at a time that confers maximum antibody protection against an anticipated seasonal surge. However, historical incidence data from a similarly seasonal respiratory virus, influenza, suggest that the presence of systematic seasonal vaccination efforts will not substantially alter the seasonality of an endemic respiratory disease. Influenza vaccines have been widely available for decades, with the center for disease control urging vaccination in the months prior to the known seasonal spikes in incidence (84). Despite this policy, the seasonal incidence patterns of influenza remain similar to those observed prior to the development of the vaccine. This robustness to vaccination timing is likely a consequence of multiple interacting factors including vaccine inequity (85), vaccine hesitation (86), and antigenic evolution of the pathogen relative to the vaccine (87). Vaccination efforts against COVID-19 face similar challenges (88–90). Consequently, it is quite possible that endemic incidence levels will approach projected seasonal trends even with the availability of vaccines, administered at appropriate times of the year, that confer effective but rapidly waning protection against SARS-CoV-2.

### Limitations

The seasonal coronavirus incidences in each location were collected in studies that monitored disease in distinct time spans and that may have been subject to a number of annually varying factors that can drive seasonal trends of respiratory infections. However, in many cases, the incidences were obtained across multiple years of sampling. For example, the Stockholm, Sweden data set (61) encompasses 2,093 samples spanning a full decade. Consequently, it is unlikely that the month-to-month average incidences of these long-term data sets are substantially affected by anomalous years. Our results project a seasonal rhythm of SARS-CoV-2 that is broadly similar to the trends observed among many major human-infecting respiratory viruses (91–93). This well-known seasonal trend toward greater respiratory incidence in the winter is typically considered to be muted in the tropics and reversed in the southern hemisphere (93, 94) and has been associated with a number of factors: temperature (19, 95, 96), humidity (97–100), solar ultraviolet radiation (101), and host behavior (102). The significance of these factors relative to each other—and whether additional factors influence seasonality of SARS-CoV-2—remains to be determined.

Across the data sets assembled for this study, there was also substantial non-temporal variance in patients who were sampled for coronavirus infection. Some data sets were largely or wholly restricted to infants or children (63), whereas others were cross-populational studies aggregating a mix of children, teenagers, and adults (24, 68). A forecast of absolute case numbers could certainly vary between cohorts (66). However, this variance in sampling should not impact our estimates of relative seasonal infection trends. This invariance in seasonal incidence arises because relative incidence in children is strongly correlated with relative incidence in other subsets of the local population (58). Any relative scale will work to reveal when higher or lower relative incidence should be expected. Indeed, the relative seasonal patterns for the long-term circulating coronaviruses from our analysis of these data sets are consistent with expectations determined for other seasonal respiratory viruses (91–93).

Our search of the literature and subsequent analyses reflect the spatiotemporal biases toward surveillance in only a few countries—aligned with broader patterns of health disparity (103–105). An expanded global surveillance of endemic seasonal coronavirus incidence—especially in the undersampled tropics and southern hemisphere—will enhance our understanding of coronavirus seasonality and facilitate preparedness. Sampling in the tropics would enable testing of the muted seasonality that appears there; sampling in the southern hemisphere would enable testing of a hypothesis of inverted seasonality compared to the northern hemisphere. Moreover, denser sampling across any areas would enable more precise regional estimates. For example, continual long-term monitoring of coronaviruses using clinical sampling (106, 107) or wastewater (108–110) could strengthen the foundation for forecasting not only long-term circulating coronavirus seasonality but also the seasonality of emergent coronaviruses such as SARS-CoV-2.

### Conclusions

Both public health interventions and evolutionary change impact whether the projected seasonality of SARS-CoV-2 will be observed. Transmission could be dampened by the acceleration of vaccination efforts around the world that, like other interventions, have the potential to disrupt erstwhile seasonality. Alternatively, the emergence of novel variants with elevated transmissibility—such as the Delta or Omicron variants (111–113)—have the potential to thwart public health efforts and impact seasonal trends. Our results suggest that surges of infection by novel SARS-CoV-2 variants will frequently coincide with anticipated surges in other seasonal endemic respiratory viruses including influenza and respiratory syncytial virus (114, 115). Our projections affirm the need for systematic, prescient public health interventions that are cognizant of seasonality.

Foreknowledge of seasonality will enable informed, advanced public health messaging regarding seasons of high concern that could help to overcome barriers of non-adherence. Even with widespread vaccination efforts, SARS-CoV-2 will join HCoV-229E, HCoV-NL63, HCoV-OC43, and HCoV-HKU1 as a coronavirus causing endemic disease (116). For epidemiological inferences such as seasonality that require long-term longitudinal data sets, evolutionary biology can provide the theoretical foundation to deliver swift, quantitative, and rigorous insight into how novel threats to human health may behave. Our approach provides guidance for myriad public health decisions as the pandemic phase of SARS-CoV-2 spread diminishes and collection of long-term data on endemic COVID-19 incidence becomes feasible.

## Data Availability

All data, inferred phylogenetic trees, imputed monthly proportions, and code underlying this study are publicly available on Zenodo: DOI: 10.5281/zenodo.10045122.

## SUPPLEMENTAL MATERIAL

Supplemental material mbio.01426-23-s0001.pdf Supplemental Materials and Methods and Figures S1–S9.

ASM does not own the copyrights to Supplemental Material that may be linked to, or accessed through, an article. The authors have granted ASM a non-exclusive, world-wide license to publish the Supplemental Material files. Please contact the corresponding author directly for reuse.
