# Supplementary material for: Seasonality of endemic COVID-19
Source: mBio. 2023 Nov 8;14(6):e01426-23. doi: 10.1128/mbio.01426-23 (PMC10746271; doi:10.1128/mbio.01426-23)
Supplement: Supplemental material — s and Methods and Figures S1–S9. [file mbio.01426-23-s0001.pdf]

Coronavirus evolution illuminates seasonality of COVID-19

Supplementary materials

Contents

Investigators … … … … … … … … … … … … … … … … … … … 02

Materials and Methods … … … … … … … … … … … … … … … … … … 03

Supplemental Figure S1 … … … … … … … … … … … … … … … … … 04

Supplemental Figure S2 … … … … … … … … … … … … … … … … 05

Supplemental Figure S3 … … … … … … … … … … … … … … 06

Supplemental Figure S4 … … … … … … … … … … … … 07

Supplemental Figure S5 … … … … … … … … … … … … … 08

Supplemental Figure S6 … … … … … … … … … … … … … … 09

Supplemental Figure S7 … … … … … … … … … … … … … … … 10

Supplemental Figure S8 … … … … … … … … … … … … … … … … 11

Supplemental Figure S9 … … … … … … … … … … … … … … … … … 12

References … … … … … … … … … … … … … … … … … … 13

Investigators

Jeffrey P. Townsend, PhD[1,2,3,4]

Hayley B. Hassler, MS[1]

April D. Lamb, MS[5]

Pratha Sha, PhD[6]

Aia Alvarex Nishio[7]

Cameron Nguyen[5]

Alexandra D. Tew BS[5]

Alison P. Galvani, PhD[6]

Alex Dornburg, PhD[5]

[1]Department of Biostatistics, Yale School of Public Health; New Haven, Connecticut 06510, USA [2]Department of Ecology and Evolutionary Biology, Yale University; New Haven, Connecticut 06525, USA 3Program in Computational Biology and Bioinformatics, Yale University; New Haven, Connecticut 06511, USA 4Program in Microbiology, Yale University, New Haven; Connecticut 06511, USA

5Department of Bioinformatics and Genomics, University of North Carolina; Charlotte, NC 28223

[6]Center for Infectious Disease Modeling and Analysis, Yale University; New Haven, Connecticut 06510, USA

7Yale College; New Haven, Connecticut

Materials and Methods

Study Design

We performed a comparative evolutionary analysis on monthly verified cases of HCoV-NL63, HCoV-229E, HCoV-HKU1, and HCoV-OC43 infection within populations across the globe. We applied ancestral and descendent states analyses on reconstructions of the evolutionary history of human-infecting coronaviruses to estimate the expected annual changes in cases at different geographic locales. These analyses provide a global-scale projection of the likely global changes of endemic seasonality for SARS-CoV-2.

Data acquisition

Phylogenetic tree topologies—Phylogenetic relationships of SARS-CoV-2 and the endemic human-infecting coronaviruses were based on data from 58 Alphacoronavirus, 105 Betacoronavirus, 11 Deltacoronavirus, and three Gammacoronavirus as analyzed in Townsend et al. (in press); Fig. 1A). These estimates of the phylogenetic topology were consistent with previous hypotheses of evolutionary relationships among coronaviruses (1–5)and were congruent across multiple methods of inference with strong (100% bootstrap) support for all nodes. Tree topologies were inferred by multiple maximum-likelihood (ML) analyses of the concatenated DNA sequence alignment, and results were robust to alternative phylogenetic likelihood search algorithms—IQ-TREE v2.0.6 (6) and RAxML v7.2.8 (7)—and to branch-length differences arising from different approaches to divergence time estimation—IQ-TREE v2.0.6 (6), Relative Times (RelTime; 8) in MEGA X v10.1.9 (9) and TreeTime v0.7.6 (10)—and to a potential history of recombination among or within genes, through phylogenetic analyses using an alignment of the putative non-recombining blocks (11). All trees from Townsend et al (in press) were pruned of SARS-CoV-1 and MERS-CoV branches because temporal trends of infection by these viruses reflect short-term outbreaks and not seasonal endemic circulation.

Seasonal infection data—We conducted a literature search using the PubMed and Google Scholar databases searching for terms related to coronavirus, seasonality, and the known seasonalendemic human-infecting coronaviruses (HCoV-NL63, HCoV-229E, HCoV-HKU1, and HCoV-OC43). Searches were conducted in English between October 2020–August 2021, using the names of each coronavirus lineage as a key term in addition to all combinations of: coronavirus, seasonality, environmental, incidence, infection, prevalence, latitude, temperature, humidity, weather, global, cases—with no language restrictions imposed. Seasonal infection data were extracted from published, peer-reviewed research papers that reported monthly or finer seasonal case data for three or more coronaviruses, spanning at least one year.

Estimating the seasonality of SARS-CoV-2

To estimate the seasonality of infections by SARS-CoV-2, we first extracted the average numbers of cases per month testing positive for HCoV-NL63, -229E, -HKU1, and -OC43 for each location. We scaled these case counts by the annual total to yield proportions of the cases sampled in each month. We then performed a phylogenetically informed ancestral and descendent states analysis, executing Rphylopars v0.2.12 (17) on the monthly proportions of cases to estimate the proportion of yearly infection by SARS-CoV-2 each month for each location, executing Rphylopars v0.2.12 (17) on the monthly proportions of cases. This approach takes known trait values (here, monthly proportions of cases for endemic coronaviruses) and applies a Brownian model of trait evolution and a phylogeny to estimate unobserved trait values for a taxon or taxa, providing best linear unbiased predictions that are mathematically equivalent to universal kriging (Gaussian process regression). Phylogenetic ancestral and descendent analyses were repeated across all topologies resulting from different inference approaches (molecular trees, relative phylogenetic chronograms, and non-recombinant alignment) to assess the impact of phylogenetic inference methods on our estimation of seasonality.

HCoV-NL63

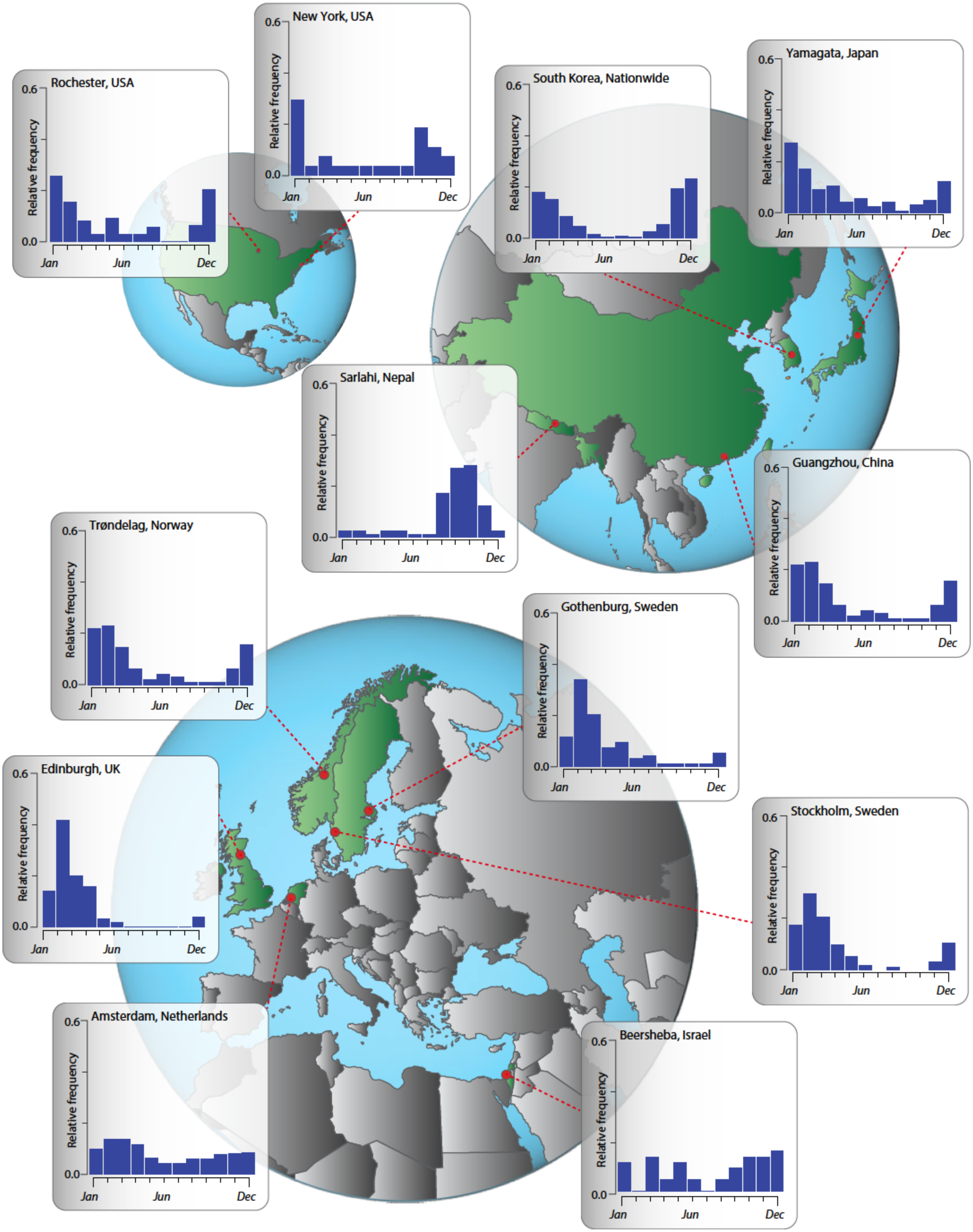

Supplementary Figure S1
Proportions of HCoV-NL63 infections by month across locations in North America, Asia, the UK, Europe, and the Middle East.

HCoV-229E

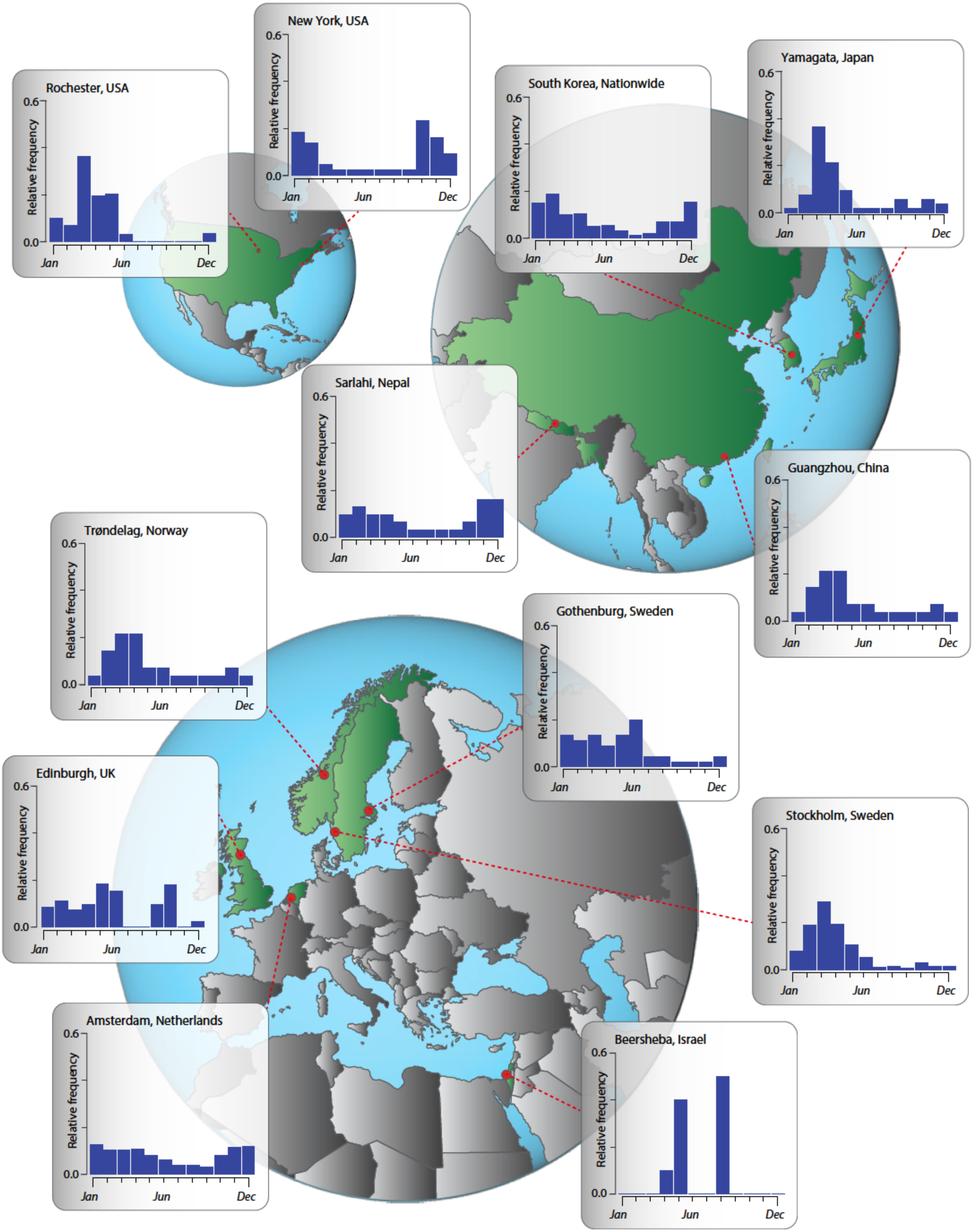

Supplementary Figure S2
Proportions of HCoV-229E infections by month across locations in North America, Asia, the UK, Europe, and the Middle East.

HCoV-HKU1

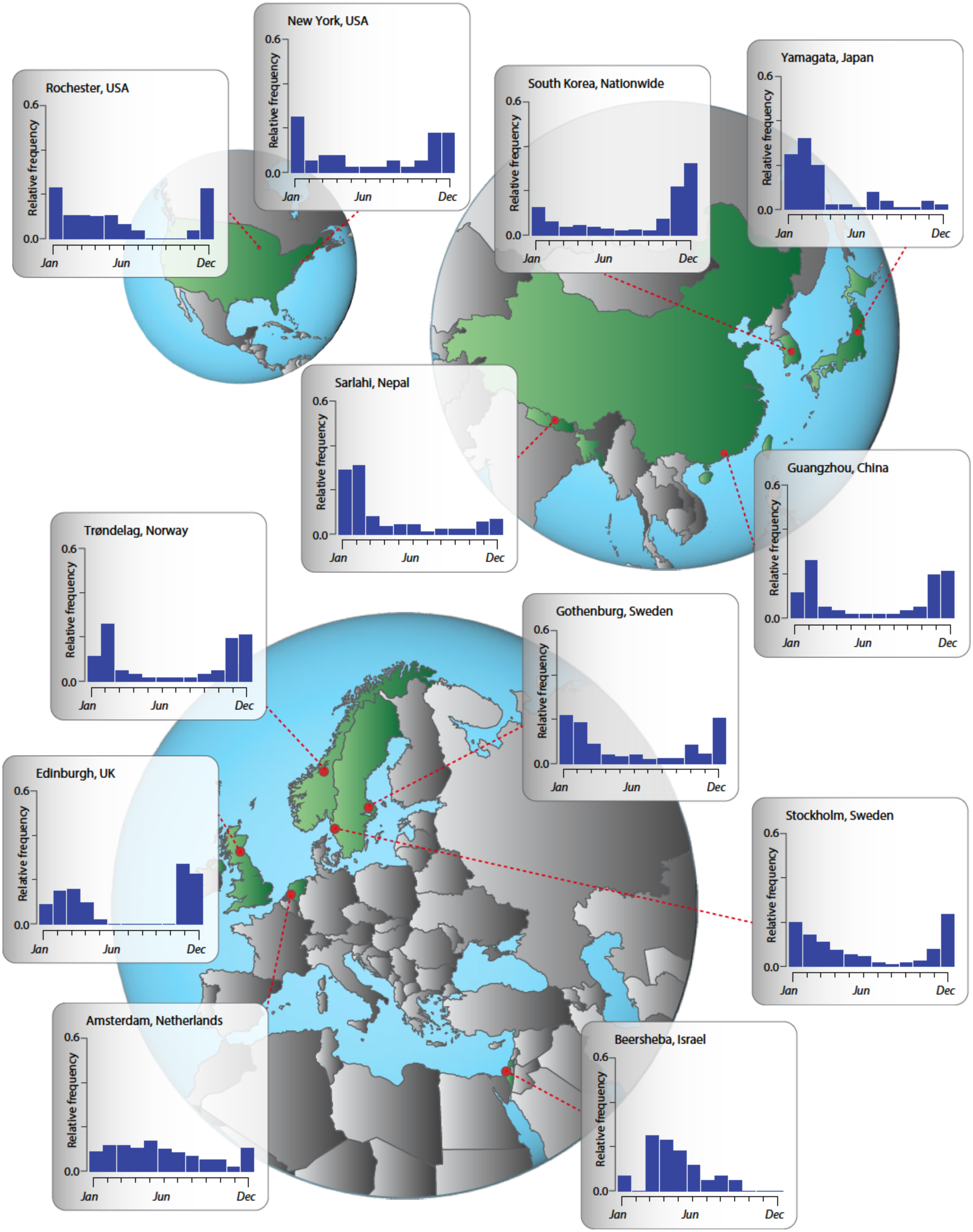

Supplementary Figure S3
Proportions of HCoV-HKU1 infections by month across locations in North America, Asia, the UK, Europe, and the Middle East.

HCoV-OC43

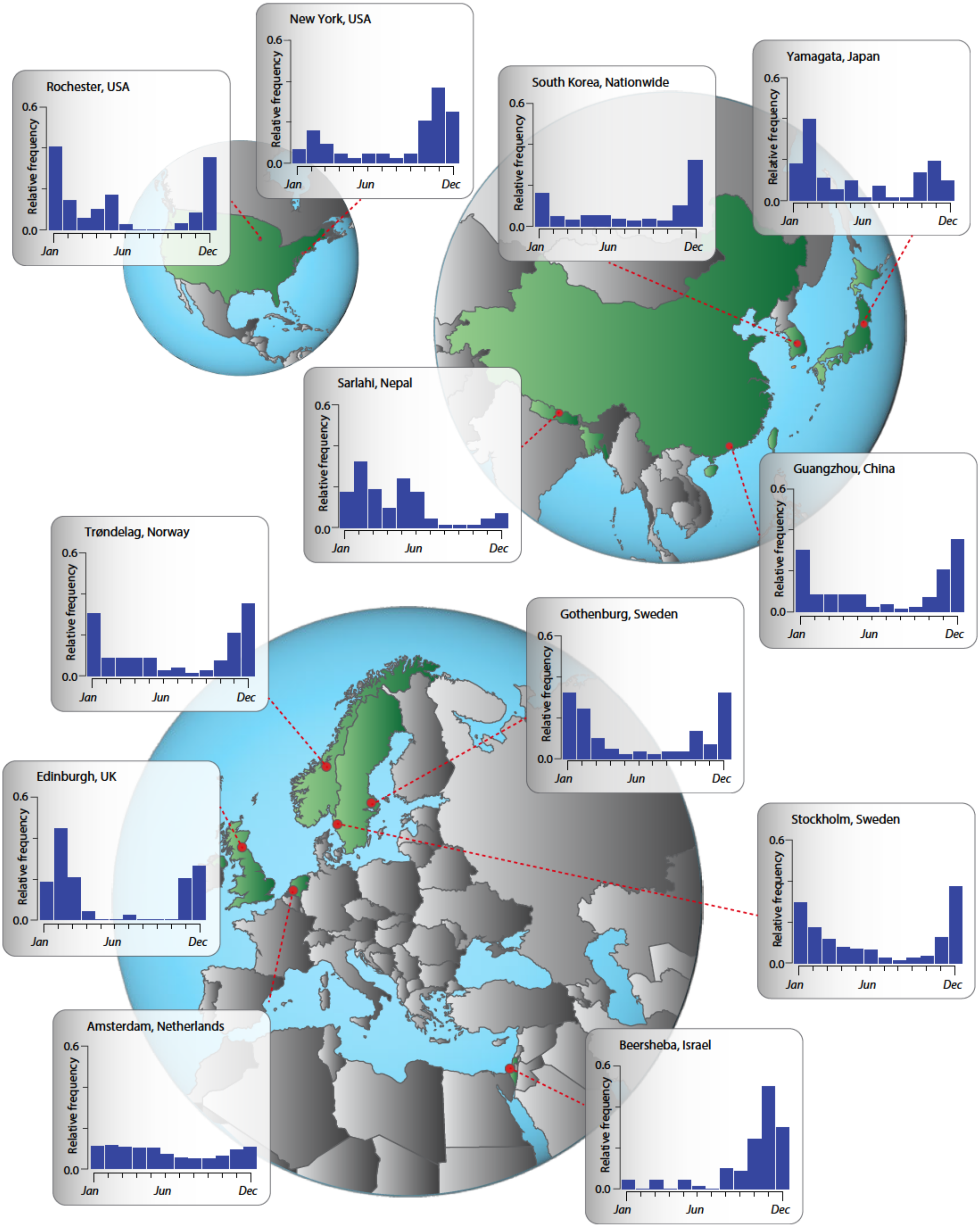

Supplementary Figure S4
Proportions of HCoV-OC43 infections by month across locations in North America, Asia, the UK, Europe, and the Middle East.

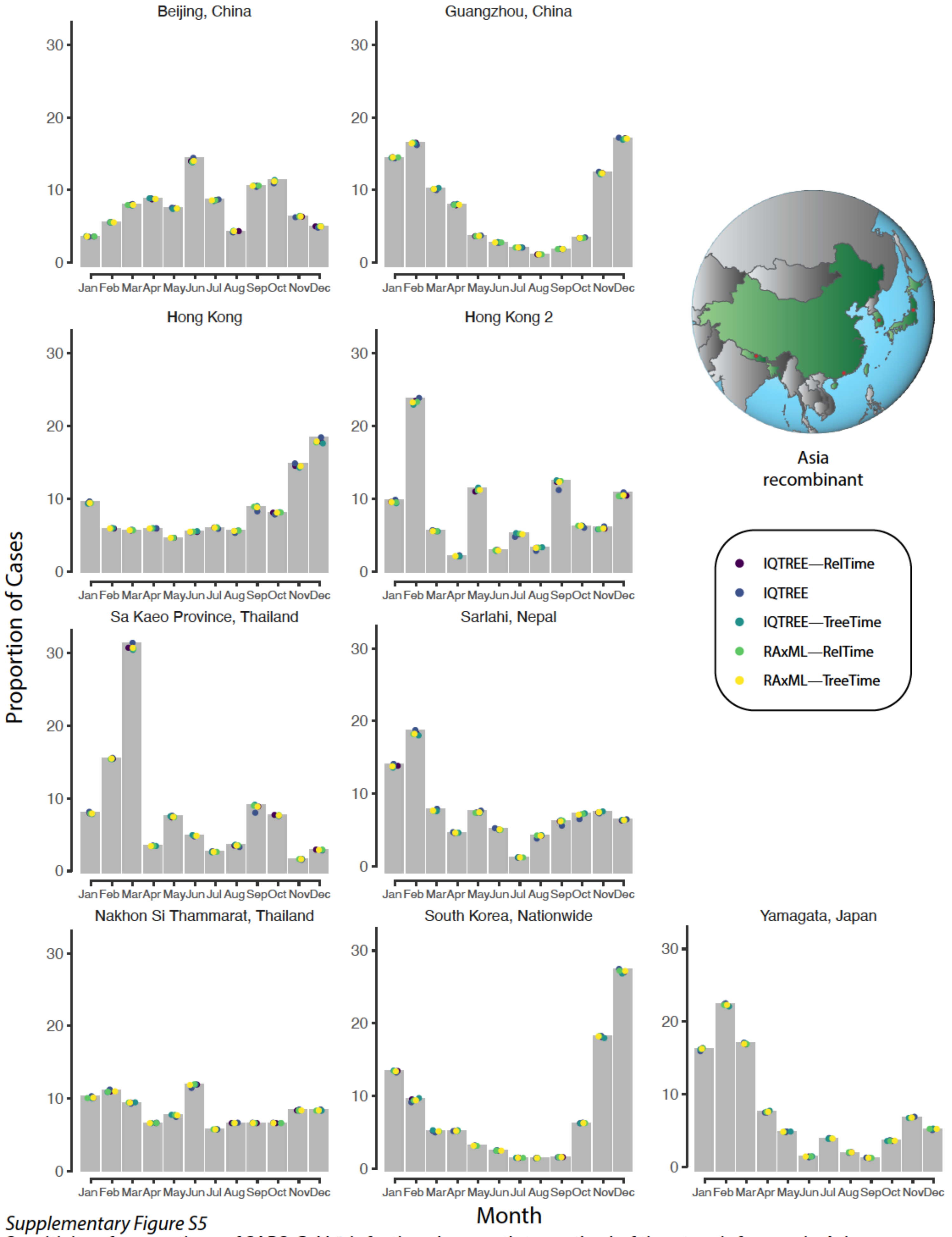

Supplementary Figure S5
Sensitivity of proportions of SARS-CoV-2 infections by month to method of time tree inference in Asia.

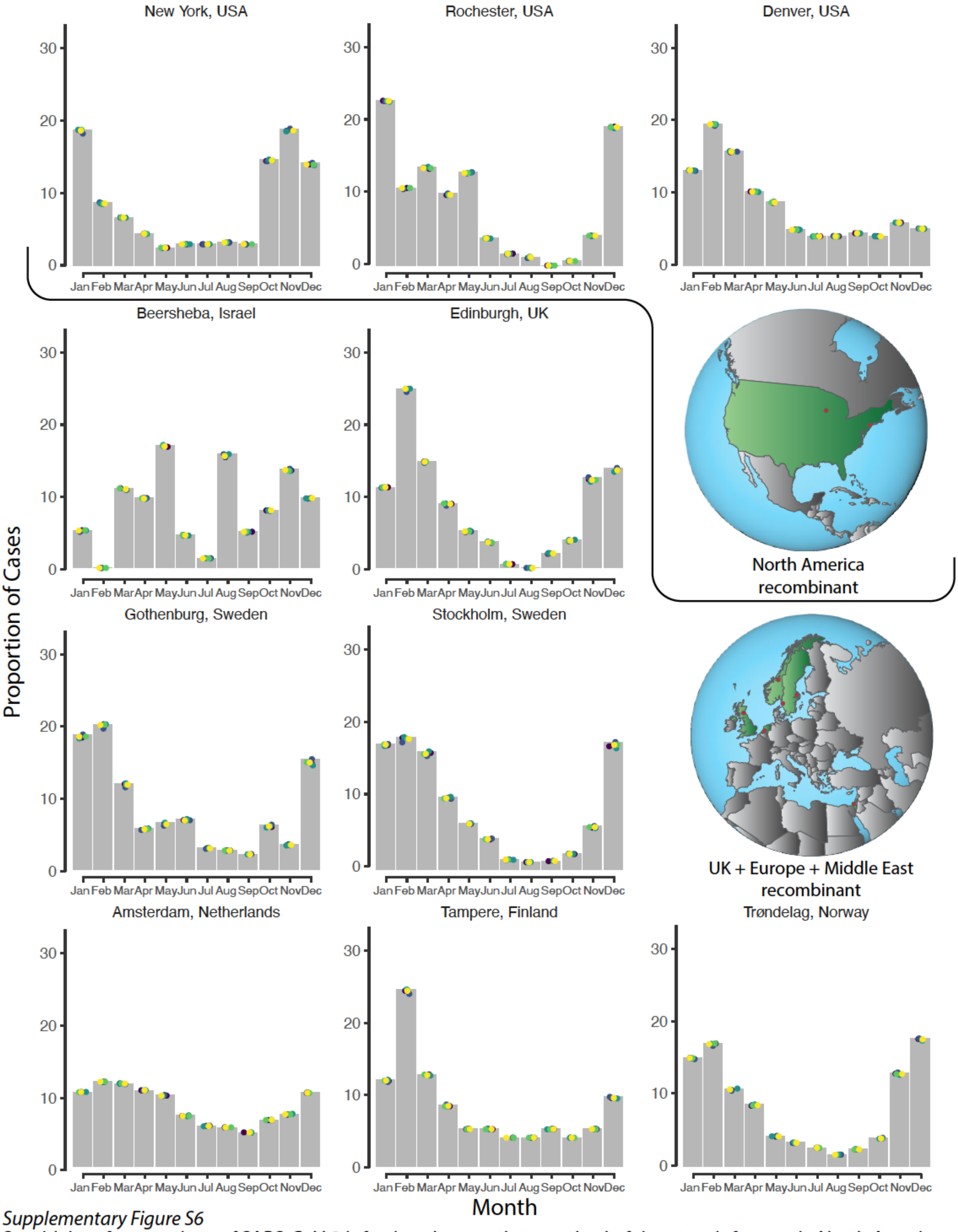

Supplementary Figure S6
Sensitivity of proportions of SARS-CoV-2 infections by month to method of time tree inference in North America, the UK, Europe, and the Middle East.

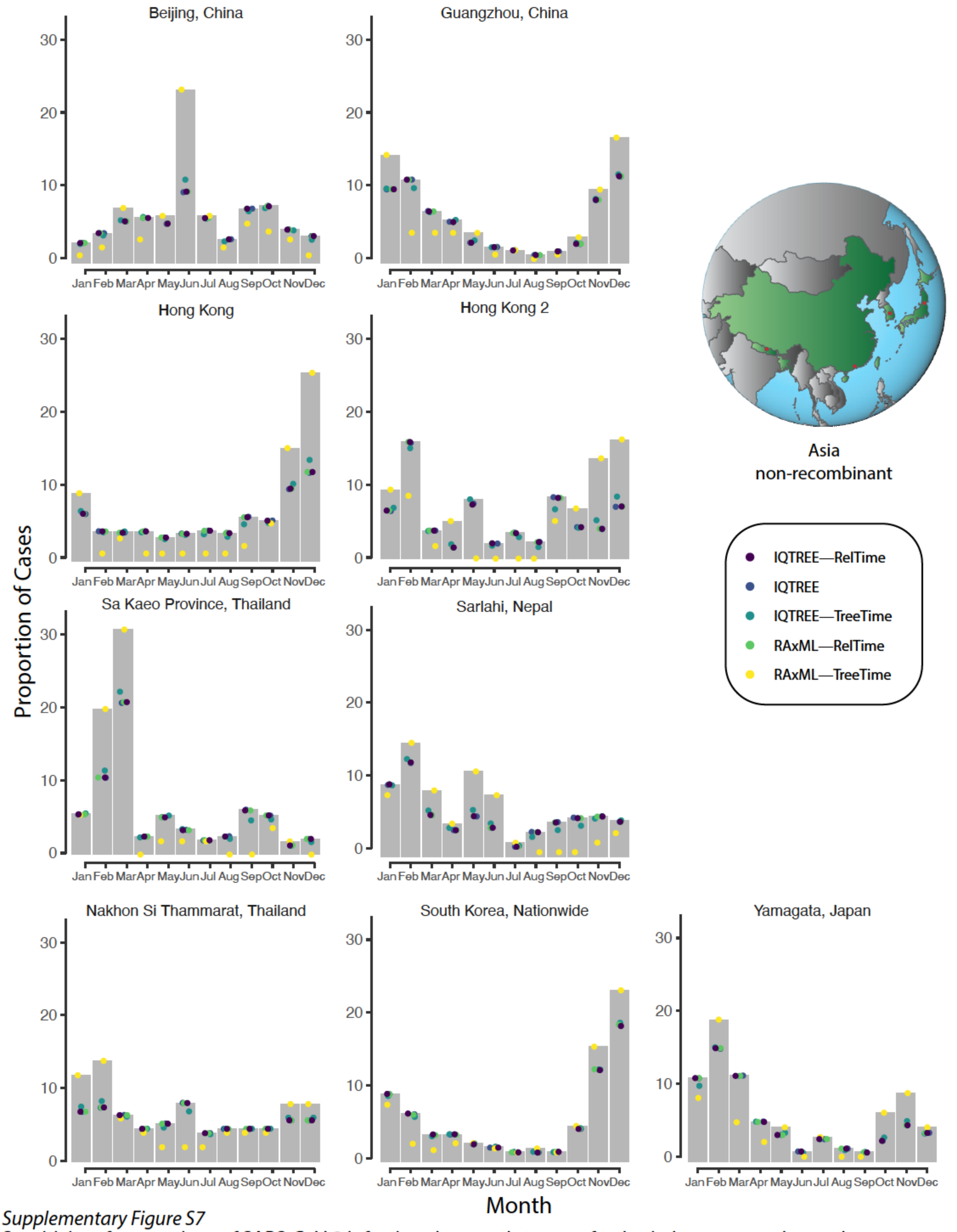

Supplementary Figure S7
Sensitivity of proportions of SARS-CoV-2 infections by month to use of only viral sequences deemed non-recombining by location in Asia.

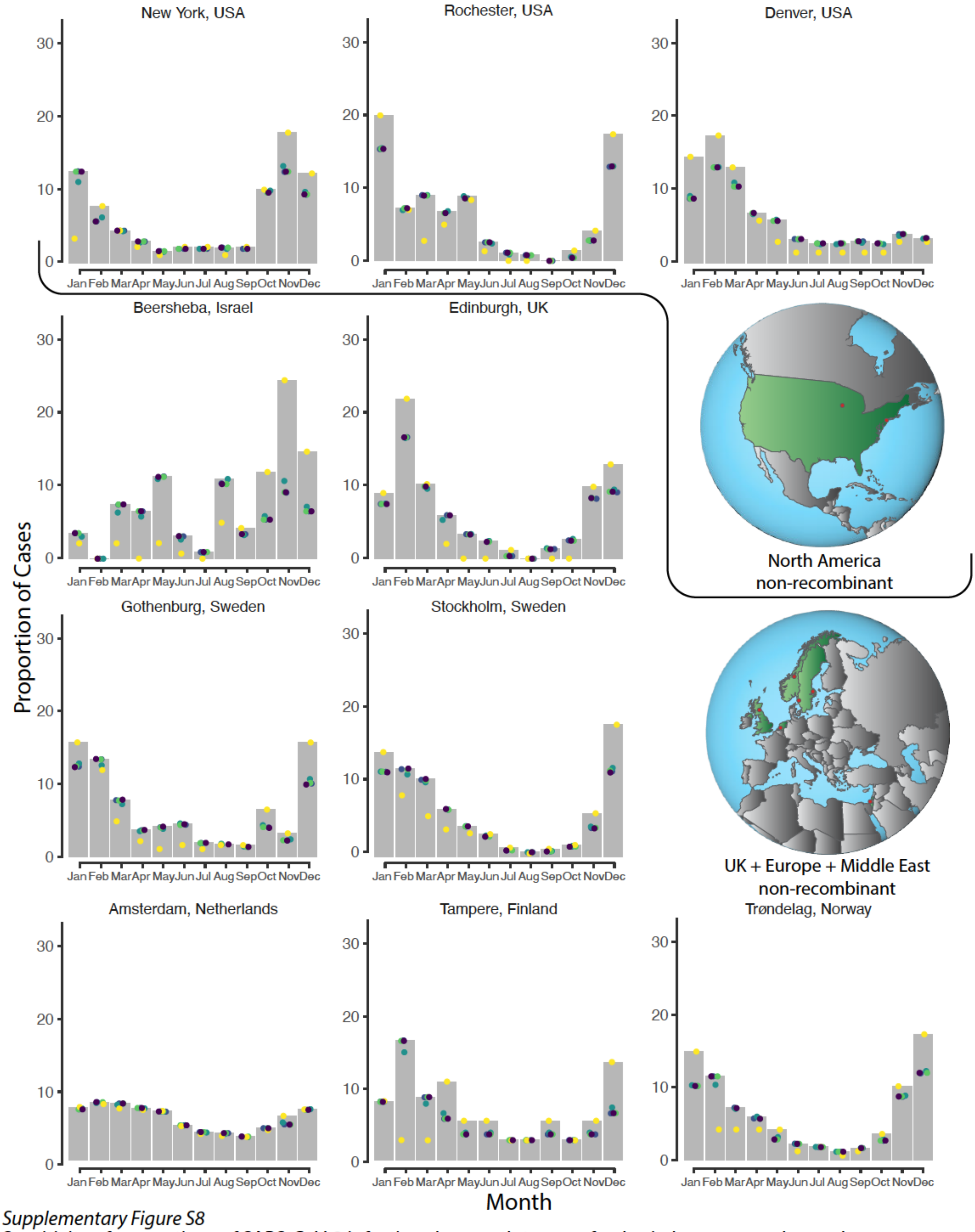

Supplementary Figure S8
Sensitivity of proportions of SARS-CoV-2 infections by month to use of only viral sequences deemed non-recombining by location in North America, the UK, Europe, and the Middle East.

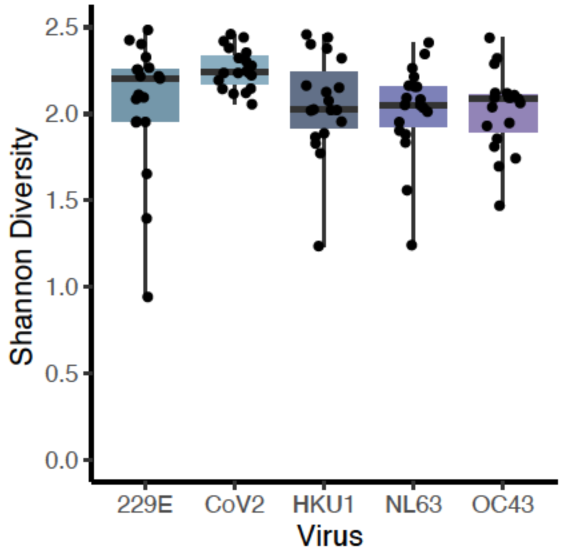

Supplementary Figure S9.
Degrees of seasonality of each coronavirus across location measured by the Shannon diversity of the proportion of yearly infection of each virus, in each month for each location

1. P. C. Y. Woo, S. K. P. Lau, C. S. F. Lam, C. C. Y. Lau, A. K. L. Tsang, J. H. N. Lau, R. Bai, J. L. L. Teng, C. C. C. Tsang, M. Wang, Others, Discovery of seven novel Mammalian and avian coronaviruses in the genus deltacoronavirus supports bat coronaviruses as the gene source of alphacoronavirus and betacoro navirus and avian coronaviruses as the gene source of gammacoronavirus and deltacoronavirus. J. Virol. 86, 3995–4008 (2012).
2. X. Fu, B. Fang, Y. Liu, M. Cai, J. Jun, J. Ma, D. Bu, L. Wang, P. Zhou, H. Wang, G. Zhang, Newly emerged porcine enteric alphacoronavirus in southern China: Identification, origin and evolutionary history analysis. Infect. Genet. Evol. 62, 179–187 (2018).
3. S. Pfefferle, S. Oppong, J. F. Drexler, F. Gloza-Rausch, A. Ipsen, A. Seebens, M. A. Müller, A. Annan, P. Vallo, Y. Adu-Sarkodie, T. F. Kruppa, C. Drosten, Distant relatives of severe acute respiratory syndrome coronavirus and close relatives of human coronavirus 229E in bats, Ghana. Emerg. Infect. Dis. 15, 1377–1384 (2009).
4. L. A. Sipulwa, J. R. Ongus, R. L. Coldren, W. D. Bulimo, Molecular characterization of human corona viruses and their circulation dynamics in Kenya, 2009–2012. Virol. J. 13, 18 (2016).
5. Coronaviridae Study Group of the International Committee on Taxonomy of Viruses, The species Se vere acute respiratory syndrome-related coronavirus: classifying 2019-nCoV and naming it SARS-CoV-2. Nat Microbiol. 5, 536–544 (2020).
6. B. Q. Minh, H. A. Schmidt, O. Chernomor, D. Schrempf, M. D. Woodhams, A. von Haeseler, R. Lan fear, IQ-TREE 2: New Models and Efficient Methods for Phylogenetic Inference in the Genomic Era. Mol. Biol. Evol. 37, 1530–1534 (2020).
7. A. Stamatakis, RAxML version 8: a tool for phylogenetic analysis and post-analysis of large phyloge nies. Bioinformatics. 30, 1312–1313 (2014).
8. K. Tamura, F. U. Battistuzzi, P. Billing-Ross, O. Murillo, A. Filipski, S. Kumar, Estimating divergence times in large molecular phylogenies. Proc. Natl. Acad. Sci. U. S. A. 109, 19333–19338 (2012).
9. S. Kumar, G. Stecher, M. Li, C. Knyaz, K. Tamura, MEGA X: Molecular Evolutionary Genetics Analy sis across Computing Platforms. Mol. Biol. Evol. 35, 1547–1549 (2018).
10. P. Sagulenko, V. Puller, R. A. Neher, TreeTime: Maximum-likelihood phylodynamic analysis. Virus Evol. 4, vex042 (2018).
11. M. F. Boni, P. Lemey, X. Jiang, T. T.-Y. Lam, B. Perry, T. Castoe, A. Rambaut, D. L. Robertson, Evolutionary origins of the SARS-CoV-2 sarbecovirus lineage responsible for the COVID-19 pandemic, doi:10.1101/2020.03.30.015008.
